# Identification of Biomarkers Associated With CD4^+^ T-Cell Infiltration With Gene Coexpression Network in Dermatomyositis

**DOI:** 10.3389/fimmu.2022.854848

**Published:** 2022-05-30

**Authors:** Peng Huang, Li Tang, Lu Zhang, Yi Ren, Hong Peng, Yangyang Xiao, Jie Xu, Dingan Mao, Lingjuan Liu, Liqun Liu

**Affiliations:** ^1^ Department of Pediatrics, The Second Xiangya Hospital of Central South University, Changsha, China; ^2^ Children’s Brain Development and Brain injury Research Office, The Second Xiangya Hospital of Central South University, Changsha, China

**Keywords:** dermatomyositis, WGCNA, CD4^+^ T cells, key gene, biomarkers, bioinformatics

## Abstract

**Background:**

Dermatomyositis is an autoimmune disease characterized by damage to the skin and muscles. CD4^+^ T cells are of crucial importance in the occurrence and development of dermatomyositis (DM). However, there are few bioinformatics studies on potential pathogenic genes and immune cell infiltration of DM. Therefore, this study intended to explore CD4^+^ T-cell infiltration–associated key genes in DM and construct a new model to predict the level of CD4^+^ T-cell infiltration in DM.

**Methods:**

GSE46239, GSE142807, GSE1551, and GSE193276 datasets were downloaded. The WGCNA and CIBERSORT algorithms were performed to identify the most correlated gene module with CD4^+^ T cells. Matascape was used for GO enrichment and KEGG pathway analysis of the key gene module. LASSO regression analysis was used to identify the key genes and construct the prediction model. The correlation between the key genes and CD4^+^ T-cell infiltration was investigated. GSEA was performed to research the underlying signaling pathways of the key genes. The key gene-correlated transcription factors were identified through the RcisTarget and Gene-motif rankings databases. The miRcode and DIANA-LncBase databases were used to build the lncRNA-miRNA-mRNA network.

**Results:**

In the brown module, 5 key genes (chromosome 1 open reading frame 106 (*C1orf106*), component of oligomeric Golgi complex 8 (*COG8*), envoplakin (*EVPL*), GTPases of immunity-associated protein family member 6 (*GIMAP6*), and interferon-alpha inducible protein 6 (*IFI6*)) highly associated with CD4^+^ T-cell infiltration were identified. The prediction model was constructed and showed better predictive performance in the training set, and this satisfactory model performance was validated in another skin biopsy dataset and a muscle biopsy dataset. The expression levels of the key genes promoted the CD4^+^ T-cell infiltration. GSEA results revealed that the key genes were remarkably enriched in many immunity-associated pathways, such as JAK/STAT signaling pathway. The cisbp_M2205, transcription factor-binding site, was enriched in *C1orf106*, *EVPL*, and *IF16*. Finally, 3,835 lncRNAs and 52 miRNAs significantly correlated with key genes were used to build a ceRNA network.

**Conclusion:**

The *C1orf106*, *COG8*, *EVPL*, *GIMAP6*, and *IFI6* genes are associated with CD4^+^ T-cell infiltration. The prediction model constructed based on the 5 key genes may better predict the level of CD4^+^ T-cell infiltration in damaged muscle and lesional skin of DM. These key genes could be recognized as potential biomarkers and immunotherapeutic targets of DM.

## Introduction

Dermatomyositis (DM) is an autoimmune inflammatory disease and a subtype of idiopathic inflammatory myopathy characterized by typical skin lesions and symmetrical proximal muscle weakness; besides, the most common complications and causes of death are interstitial lung disease (ILD) and malignant tumors (1–3). According to the current internationally recognized diagnostic criteria for DM, such as the Bohan and Peter criteria, characteristic skin features, including heliotrope rash across the periorbital, Gottron’s sign, V-neck sign, and shawl sign, are indispensable for the diagnosis of DM (4). Although muscle involvement is common, it often occurs months or years later than skin damage, and approximately 10%–20% of DM characterized by typical skin manifestations with subtle or no muscle involvement is defined as clinically amyopathic dermatomyositis (CADM) (5, 6). Therefore, early diagnosis of DM is relatively difficult if patients lack characteristic skin lesions. Cutaneous lesions are closely associated with the disease activity and prognosis of DM, and some atypical cutaneous manifestations can be used to predict the possibility of current or future systemic diseases in DM patients. For example, mechanic’s hands are related to an increased risk of ILD in patients with DM, skin necrosis is related to an increased risk of malignant tumors in patients with DM, and skin ulceration is linked to the low survival rate of DM patients (7, 8). Due to the lack of the ability to identify atypical skin lesions of DM, patients tend to miss or delay the optimal treatment time. In addition, the difficulty of treating DM lies in that the symptoms of myositis may be significantly improved after drug treatment, but the skin lesions will still be recurrent, resulting in changes in appearance, persistent itching, and light sensitivity, which seriously affect the quality of life of DM patients (9, 10). Most importantly, the targeted therapeutic drugs for skin lesions of DM, such as the application of topical corticosteroids, systemic immunosuppressants, or biological agents, may be of some efficacy, but the efficacy is variable, lacks prospective studies, and is accompanied by many side effects (9, 10). Notably, among all types of DM, CADM has the lowest survival rate (7). All of these challenges indicate that it is urgent to develop more effective and safer novel drugs for treating skin lesions in patients with DM. Further understanding of the molecular pathogenesis of skin inflammatory injury in DM may reveal better molecular markers and more effective therapeutic targets.

Ahmed et al. pointed out that skin biopsy was sufficient to diagnose DM without muscle biopsy in the presence of the characteristic skin manifestations and muscle symptoms of DM (11). Therefore, the biopsy of skin lesions is important for the early diagnosis of DM. The histopathological features of DM skin lesions are often manifested as interface inflammatory infiltration, perivascular inflammatory infiltration, increased apoptosis of keratinocytes in the epidermis basal layer, mucin deposition in the dermis, vacuolization of basal cells, keratinizing disorder, endothelial cell damage, etc. (12). The pathogenesis of skin inflammatory injury in DM is still unclear and may involve cellular immunity, mechanical stress, sunlight exposure, etc. (13). Multiple studies have observed that CD4^+^ T lymphocytes are the most important inflammatory infiltrating cells in the skin lesions of DM, mainly distributed at the dermal–epidermal junction around the blood vessels (14–16). Most of the infiltrating CD4^+^ T lymphocytes express CD40L, indicating that activation of CD4^+^CD40L^+^ T cells may be the main mechanism leading to the characteristic skin immune damage of DM (16). CD4^+^ T lymphocytes and activated CD4^+^ memory T cells in skin lesions are positively correlated with the area and severity index score of DM skin lesions (14). Maddukuri et al. found that compared with skin from the healthy controls and peripheral blood mononuclear cells (PBMCs) of patients with DM, the inflammatory infiltration of CD4^+^ T lymphocytes in the skin lesions in DM patients increased, and CD4^+^ T lymphocytes significantly expressed cannabinoid type 2 receptor (CB2R) and also produced interleukin (IL)-4, IL-31, interferon (IFN)-γ, and IFN-β. Notably, after treatment with lenabasum (a CB2R antagonist), the infiltration of CD4^+^ T lymphocytes, as well as the expression of CB2R, IL-31, IFN-γ, and IFN-β were all downregulated in the skin lesions, but this phenomenon was not observed in the PBMCs of DM patients (9). Their study not only indicated that CB2R regulating CD4^+^ T-cell–mediated immune inflammation is a specific skin lesion mechanism of DM but also suggested that although CD4^+^ T-lymphocyte infiltration is dominant in the lesional skin, damaged muscle, and peripheral blood of DM, there may be some differences in the immune regulatory mechanisms. DM is regarded as a CD4^+^ T-cell–driven disease, and CD4^+^ T cells might participate in the occurrence and development of DM in the following ways: mediating the growth, proliferation, classification, and transformation of B cells, indirectly participating in the production of myositis-specific autoantibodies (MSAs) and myositis-associated autoantibodies (MAAs), and the differentiation of T helper (Th) cells (17–21). However, the mechanism of CD4^+^ T-lymphocyte infiltration in DM remains unclear. Therefore, the identification of novel biomarkers associated with CD4^+^ T-cell infiltration may contribute to the exploration of the immune infiltration mechanism of DM, and plays an important guiding role for the early diagnosis, the evaluation of prognosis, and the discovery of new therapeutic targets of DM, especially for DM with skin lesions.

With the rapid development of bioinformatics, increasing online tools are being applied to find novel biomarkers, particularly those correlated to immunity. Weighted gene coexpression network analysis (WGCNA) is a powerful tool for the identification of biologically relevant associations between phenotypes and gene modules (22). It has been extensively utilized for the recognition of new biomarkers and therapeutic targets for diverse diseases at the transcriptional level (23, 24). Cell type identification by estimating relative subsets of RNA transcripts (CIBERSORT) is a deconvolution algorithm to analyze gene expression data of different immune cells (25). The least absolute shrinkage and selection operator (LASSO) regression is an analytical method that prevents overfitting through L1 regularization and is used to identify the key genes with high forecast accuracy (26).

In this study, gene expression profiles of lesional skin samples from DM patients and the normal control group were downloaded from the Gene Expression Omnibus (GEO) database. The CIBERSORT algorithm was performed to quantify the proportion of infiltrating immune cells in DM lesional skin. The infiltration scores of seven T-cell subtypes were selected as WGCNA phenotypic data for the WGCNA analysis to recognize coexpressed genes and explore the interrelation between gene modules and phenotypes. Gene Ontology (GO) enrichment analysis and Kyoto Encyclopedia of Genes and Genomes (KEGG) pathway analysis were applied to further assess the underlying function of the key gene module. By the LASSO method, the key genes were identified and a prediction model was constructed. We validated the performance of the constructed prediction model on another skin biopsy dataset and a muscle biopsy dataset from DM patients. The key gene expression levels of affected skin in DM, the relationship between the key genes and CD4^+^ T-cell infiltration, and the interactions between the key genes were analyzed. To explore the underlying molecular mechanisms that the key genes might be involved in, we performed gene set enrichment analysis (GSEA), researched the relationship between the key genes and disease-regulating genes, and built a network of transcriptional regulators. To gain insight into the upstream regulatory sites of the key genes, we established a long noncoding RNA (lncRNA)-microRNA (miRNA)-messenger RNA (mRNA) network, based on the competing endogenous RNA (ceRNA) theory. From what we know, this is the first time that CIBERSORT, WGCNA, and LASSO methods have been used in combination to identify novel biomarkers associated with CD4^+^ T-cell infiltration of DM, construct a prediction model evaluating CD4^+^ T-cell infiltration, and investigate the regulatory mechanisms of the key genes.

## Materials and Methods

### Data Download

**Figure 1:**
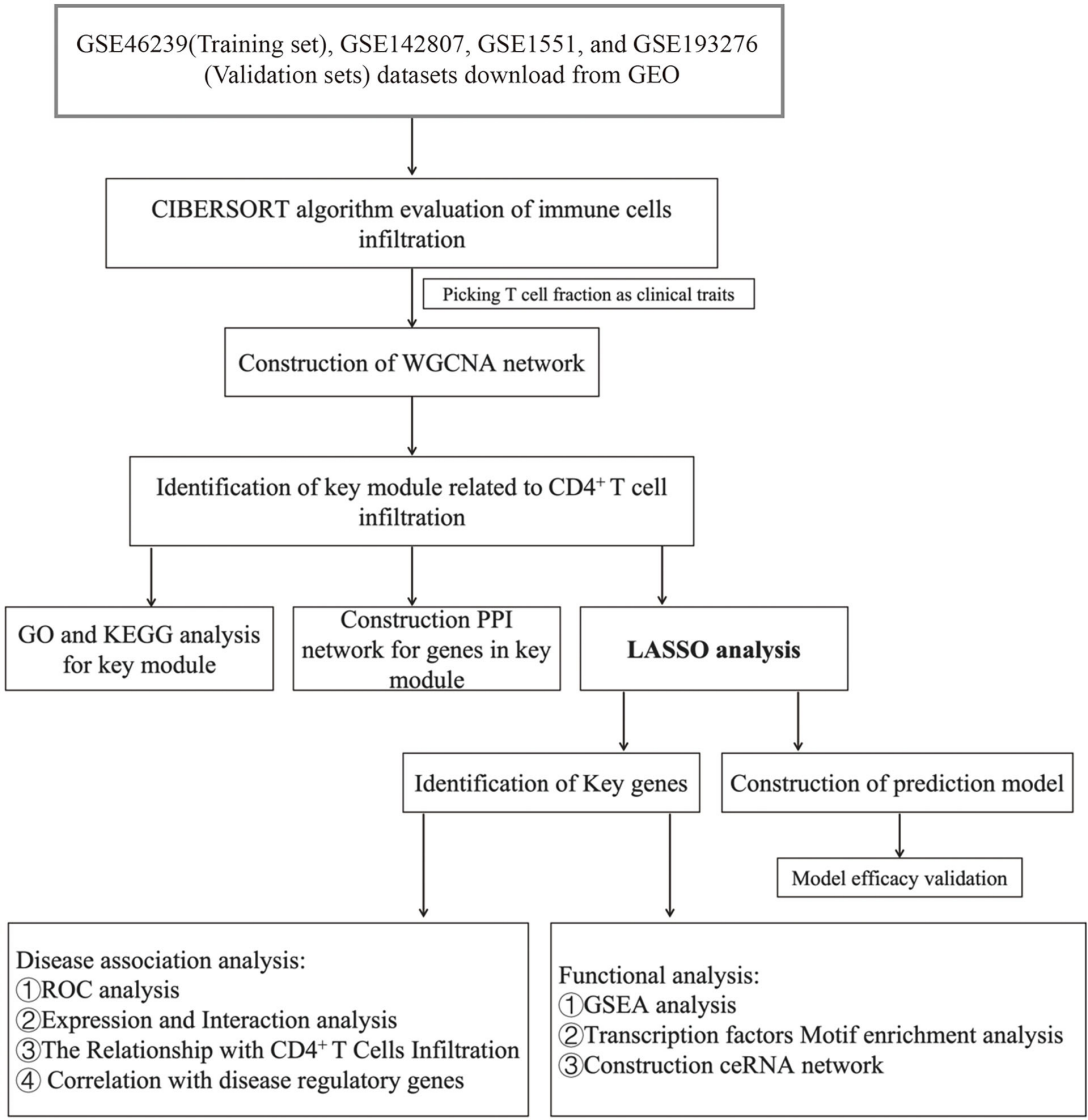
The workflow of this study.

We downloaded GSE46239, GSE142807, GSE1551, and GSE193276 datasets as Series Matrix Files from the NCBI GEO public database. The GSE46239 dataset (as a training set) was annotated by the GPL570 platform and involved the expression profile data from 52 samples of skin biopsies, including 48 DM patients and 4 normal samples (**Supplementary Table S1**). The GSE46239 dataset was chosen as the training set as it has a larger sample size compared to other skin biopsy datasets, with better statistical representation. GSE142807 was annotated by GPL17692 and consisted of the expression profile data from 48 skin biopsies, including 43 DM patients and 5 normal samples (**Supplementary Table S2**). GSE1551 was annotated by GPL96 and was composed of the expression profile data from 23 muscle biopsies of 13 DM patients and 10 normal samples (**Supplementary Table S3**). GSE193276 was annotated by GPL16791 and comprised the expression profile data from 14 skin biopsies of 7 DM patients before and after treatment (**Supplementary Table S4**). GSE142807, GSE1551, and GSE193276 were used as validation sets. **Figure 1** presents the workflow of this study.

### Immune Cell Infiltration Analysis

The CIBERSORT algorithm is commonly utilized to evaluate the types of immune cells in the microenvironment. This algorithm utilizes the gene expression values, consisting of 547 genes, to evaluate the proportion of 22 different infiltrating immune cells. In this study, we applied the CIBERSORT-R package to assess the relative percentage of immune cell infiltration at the site of lesional skin in DM. In addition, the Spearman correlation analysis was performed on the immune cell infiltration levels and gene expression levels.

### Coexpression Network Construction and Identification of Key Gene Module

The transcriptional data of all genes were extracted from the GSE46239 dataset. The gene coexpression network was constructed by using the WGCNA-R package, and the top 5,000 genes were filtered out for further analysis based on the algorithm. The function “sft$powerEstimate” determines the soft-thresholding value *β*; we set the *β* to 5. Firstly, the expression level of an individual transcript similarity matrix was transformed into an adjacency matrix, and the topological overlap matrix (TOM) was generated from the adjacency matrix to estimate the network connectivity. Secondly, the clustering tree was constructed by TOM and average linkage hierarchical clustering. One branch of the tree represents one gene module, and one color represents one module. In accordance with the weighted correlation coefficients, the genes were clustered based on their expression patterns, and genes with similar expression patterns were classified into one module. All genes were divided into several gene modules in the above way. Module eigengene (ME) is viewed as the main component of each gene module. For that reason, MEs can reflect the level of gene expression in the module. The correlation between gene modules and CD4^+^ T-cell infiltration was evaluated through the Pearson correlation test in WCGNA. Eventually, the gene module most significantly related to CD4^+^ T-cell infiltration was identified as the key gene module for further analysis.

### Functional Enrichment Analysis of Key Gene Module

To understand the biological functions and pathways of the key gene module, GO functional enrichment and KEGG pathway analyses were performed using Metascape (http://metascape.org). The enrichment cutoff used in this study was set to Min overlap ≥3 and *p* ≤ 0.01.

### Construction and Validation of Prediction Model

The LASSO regression was applied to establish the prediction model based on all genes in the key gene module. After enrolling each gene expression value, we calculated the formula for the risk score of each sample in the training set. The risk score formula was established by weighting the estimated regression coefficients in the LASSO analysis. We used the median risk score as the cutoff point and drew the receiver operating characteristic (ROC) curves to evaluate the model’s accuracy. Subsequently, the prediction model was validated by the GSE142807 and GSE1551 datasets. We used the “Circlize package” and “corrplot package” to analyze key gene correlation visualized the result using Circos analysis.

### Gene Set Enrichment Analysis

GSEA is the process of sorting genes based on the expression difference of genes from two disparate samples using a predefined gene set (according to the KEGG database) and then determining if the predefined set of genes is enriched at the top or bottom of the sorting list. To explore the underlying molecular mechanisms for the involvement of the key genes, the GSEA software (https://www.broadinstitute.org/gsea) was employed to investigate the differences in the KEGG pathways between the high- and low-expression groups of key genes. The number of the permutations was set to 1,000, and the type of the permutations was set to phenotype. The absolute value of the normalized enrichment score (NES) >1, the false-positive rate (FDR) *q*-value <0.25, and the nominal *p-*value <0.05 were deemed as the significantly enriched pathways.

### Enrichment Analysis for Transcription Factors of Key Genes

Transcription factors (TFs) participate in the initial process of eukaryotic gene transcription and can be classified into two types according to their mechanism of action (27). The first type is general TFs assembled with RNA polymerase II to form the transcription initiation complex. Under these conditions, the transcription can start at the correct location (27). The second type is *cis*-acting elements, including promoters, enhancers, and so on, involved in the regulation of gene expression (27). In this study, TF enrichment analysis was performed to identify key TFs related to the key genes. This process used the Gene-motif rankings and annotation of TFs by motifs based on the “R package RcisTarget”. The GeneCards database (https://www.genecards.org) was used to identify the regulatory genes of DM. The relationship between the key genes and regulated genes was also analyzed.

### Construction of lncRNA-miRNA-mRNA (ceRNA) Regulatory Network

MiRcode (http://mircode.org/) was performed to forecast the miRNAs of the key genes and establish the pairs of miRNA-mRNA. The DIANA-LncBase (https://diana.e-ce.uth.gr/lncbasev3/home) was applied to predict the lncRNAs through the known miRNAs and establish the pairs of lncRNA-miRNA. The commonly identified mRNAs were then incorporated. Based on the lncRNA-miRNA-mRNA interactions, a ceRNA network was built by applying the Cytoscape software. The top 5 lncRNAs with the highest connectivity were identified using the “table” function.

### Statistical Analysis

Statistical analysis was performed using R version 4.0. All statistical tests were two-sided and *p*-values of <0.05 were considered as statistically significant differences.

## Results

### Results of Immune Cell Infiltration

**Figure 2:**
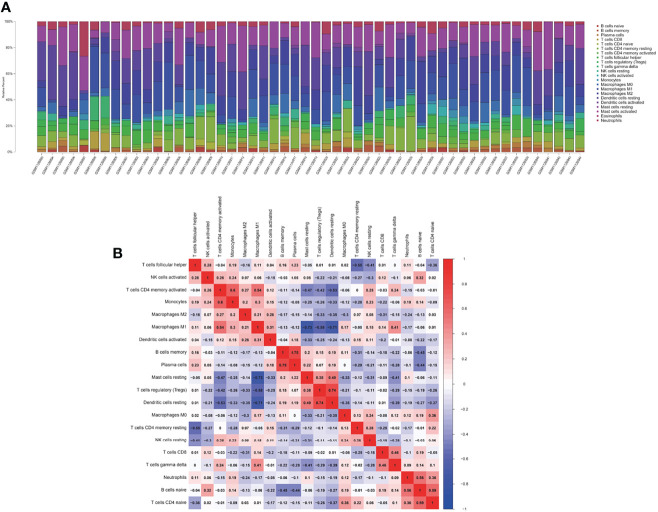
The landscape of immune infiltration between the DM and normal groups. **(A)** The cumulative histogram indicates the relative proportions of 22 immune cells. **(B)** The heatmap shows the correlation in the infiltration of 22 immune cell type proportions. Colored squares represent the strength of the correlation; the red color represents positive correlation, and the blue color represents negative correlation. The deeper the color, the stronger the correlation.

We assessed the relative abundance of immune cell infiltration of every sample through the CIBERSORT algorithm. The cumulative histogram shows the relative fractions of 20 immune cell subtypes (**Figure 2A**). The heatmap of the correlation between immune cells is presented in **Figure 2B**.

### Results of the WGCNA Analysis

**Figure 3:**
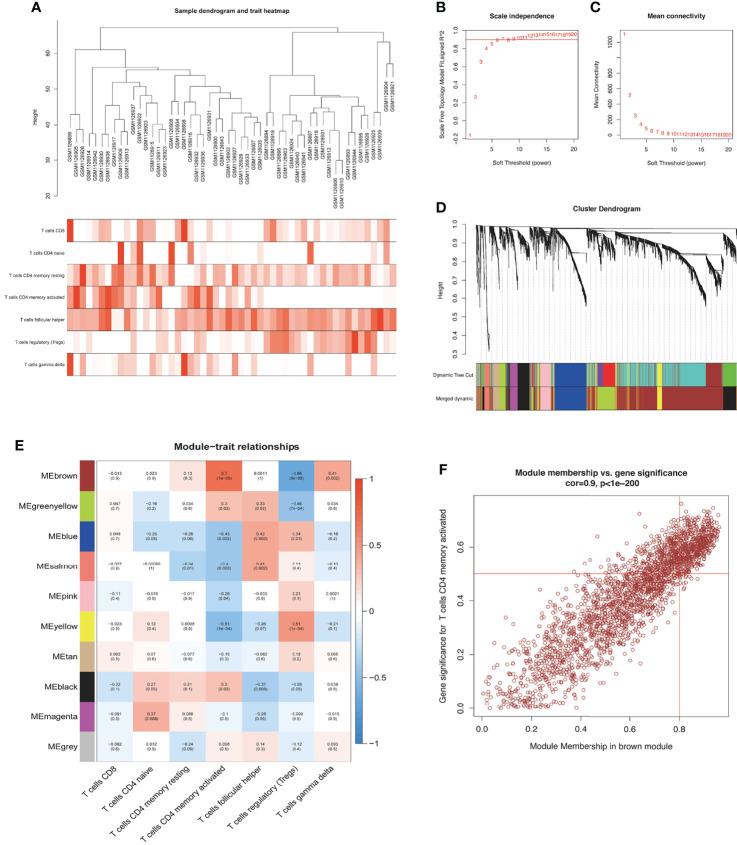
Identification of key module. **(A)** Sample dendrogram and trait heatmap. A branch indicates a single sample in our training set. **(B, C)** The process of *β* selection, the scale-free fit index, and the average connectivity for different *β*. **(D)** Cluster dendrogram. A color branch of the cluster tree represents a coexpression module. The two-colored rows below the cluster tree represent the primitive module and coalesced module. **(E)** Heatmap shows the correlations of ME with T-cell infiltration. The background color of single cells indicates the correlation strength. The red color represents positive correlation with phenotypic trait; the blue color represents negative correlation. The number in each cell indicates the correlation coefficient (*R*), and the *p*-value (in parentheses) represents correlation significance (*p* < 0.01 indicated the significant correlation). ME, module eigengene. **(F)** The correlation between ME in the brown module and T-cell CD4 memory activated (Cor = 0.9, *p* < 1e−200) (*p* < 0.01 indicated the significant correlation).

According to CIBERSORT, the infiltration fractions of seven T-cell subtypes in each sample were used as the trait data for the WGCNA analysis. Samples in the GSE46239 dataset were clustered by calculating average linkage and Pearson’s correlation coefficient. A gene coexpression network was established by the expression values of the top 5,000 genes using the WGCNA-R package, and a sample dendrogram and trait heatmap were constructed (**Figure 3A**). The power of *β* = 5 (scale-free *R* ^2^ = 0.9) was chosen as the soft-thresholding parameter to construct a scale-free network based on the scale-free topological criteria (**Figures 3B, C**). After building the hierarchical clustering tree by a dynamic hybrid cutting method, ten gene coexpression modules were generated (black module, blue module, brown module, green–yellow module, grey module, magenta module, pink module, salmon module, tan module, and yellow module) (**Figure 3D**). Gene names in each module are presented in **Supplementary Table S5**. Correlation analysis between gene modules and phenotypes showed that the brown module exhibited the highest correlation with CD4^+^ T cells (correlation coefficient (Cor) = 0.7, *p* = 1e−08) (**Figure 3E**). Also, module membership (MM) in the brown module presented a significantly positive correlation with T-cell CD4 memory activated (Cor = 0.9, *p* < 1e−200) (**Figure 3F**). We selected the brown module as the key module for subsequent analysis to explore the underlying functions and mechanisms of these genes driving CD4^+^ T-cell infiltration in DM.

### Functional Enrichment Analysis Results of Key Gene Module

**Figure 4:**
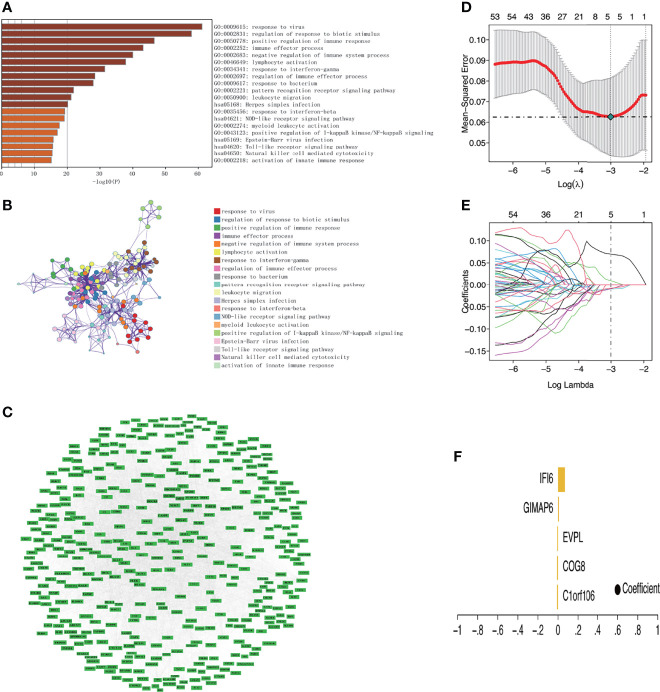
Functional enrichment analysis of key module and identification of key genes related to DM. **(A)** Bar chart of the top 20 enriched terms (The enrichment cutoff was set to Min overlap ≥3 and *p* ≤ 0.01). **(B)** Network diagram showing the enriched terms. Each enrichment term is a node; nodes with the same color share the same cluster ID. **(C)** Protein–protein interaction network of genes in the brown module. **(D)** The confidence interval of each lambda. The horizontal axis shows the logarithm of the lambda, the vertical axis shows mean-squared error. **(E)** The distribution of the LASSO coefficient. Each color line shows the changing tendency of each gene coefficient chosen by the LASSO algorithm. The horizontal axis shows the log value of lambda, the vertical axis shows the coefficient corresponding to lambda, and the numeral on the upmost axis shows the number of genes whose coefficient is not zero at different log lambda values. **(F)** Five genes were screened out by LASSO regression and their coefficient.

GO enrichment and KEGG pathway analyses identified the top 20 terms enriched in the brown module, which were immune-related terms. Among them, the four terms with the highest fold enrichment were response to the virus, regulation of response to biotic stimulus, positive regulation of immune response, and immune effector process (**Figure 4A**). **Supplementary Table S6** shows the 50 representative genes enriched by the KEGG pathways. We selected the most representative terms from each of the 20 clusters to construct a network layout (**Figure 4B**). The interaction network of all genes in the brown module was established through the string online database (https://string-db.org) and visualized using Cytoscape (**Figure 4C**).

### Construction and Validation of Prediction Model and Identification of Key Genes

**Figure 5:**
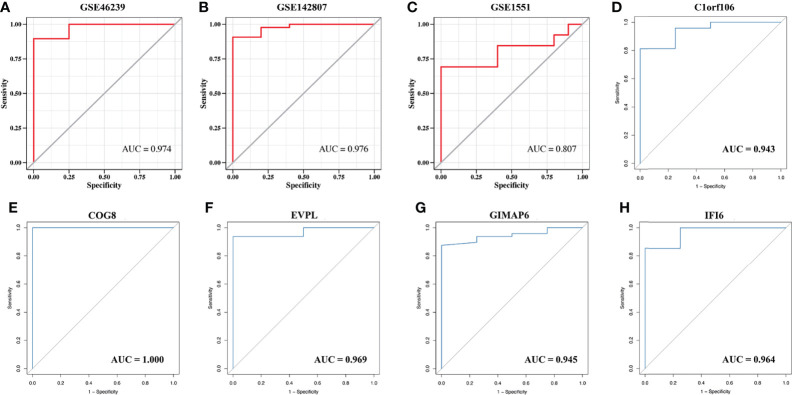
The validation of the model predictive efficacy and the evaluation of the independent predictive performance of key genes. **(A)** ROC curve analysis of the training set, AUC = 0.974. **(B)** ROC curve analysis of the GSE142807 validation set, AUC = 0.976. **(C)** ROC curves analysis of the GSE1551 validation set, AUC = 0.807. **(D–H)** ROC curve analyses respectively of *C1orf106* (AUC = 0.943), *COG8* (AUC = 1.000), *EVPL* (AUC = 0.969), *GIMAP6* (AUC = 0.945), and *IFI6* (AUC = 0.964).

The strongly connected genes in the brown module may be the underlying key factors driving the infiltration of CD4^+^ T cells. A total of 506 candidate key genes were identified from the brown module based on the cutoff standard (module membership >0.8 and gene significance >0.5). Feature selection was conducted by LASSO regression in candidate key genes. Eventually, 5 genes that can be used as characterized genes of DM were identified, which were chromosome 1 open reading frame 106 (*C1orf106*), component of oligomeric Golgi complex 8 (*COG8*), envoplakin (*EVPL*), GTPases of immunity-associated protein family member 6 (*GIMAP6*), and interferon-alpha inducible protein 6 (*IFI6*) (**Figures 4D–F**). We referred to these five genes as key genes. The prediction model was established according to the LASSO algorithm based on the expression levels of the key genes and the regression coefficients of LASSO. The risk score formula was: risk score = *C1orf106* × (-0.00944194461775629) + *COG8* × (-0.00881190286420128) + *EVPL* × (-0.0075305737266484) + *GIMAP6* × 0.00937653812406344 + *IFI6* × 0.0695325226398151. We found that the gene prediction model constructed by the 5 genes has a better predicted performance in the training set (the dataset of skin biopsies), with an area under the curve (AUC) of 0.974 (**Figure 5A**). The prediction model was verified in the GSE142807 validation set (another dataset of skin biopsies) with an AUC of 0.976 (**Figure 5B**). To understand whether this model also presents a good predictive performance in muscle biopsy tissue from DM patients, we validated the model performance in the GSE1551 dataset with an AUC of 0.807 (**Figure 5C**). The above results demonstrated that the prediction model has better stability and prediction ability in both the skin and muscle biopsy datasets. On the basis, we further evaluated the predictive value of each key gene through the ROC curve, and the results showed that the AUC values of the 5 key genes were as follows: 0.943 (*C1orf106*), 1.000 (*COG8*), 0.969 (*EVPL*), 0.945 (*GIMAP6*), and 0.964 (*IFI6*), respectively (**Figures 5D–H**). The results described above demonstrated that the prediction model based on the training set had superior accuracy, and the 5 key genes could well predict the inflammatory infiltration level of CD4^+^ T cells in DM patients.

### Expression and Interaction of Key Genes and the Relationship With CD4^+^ T-Cell Infiltration Level

**Figure 6:**
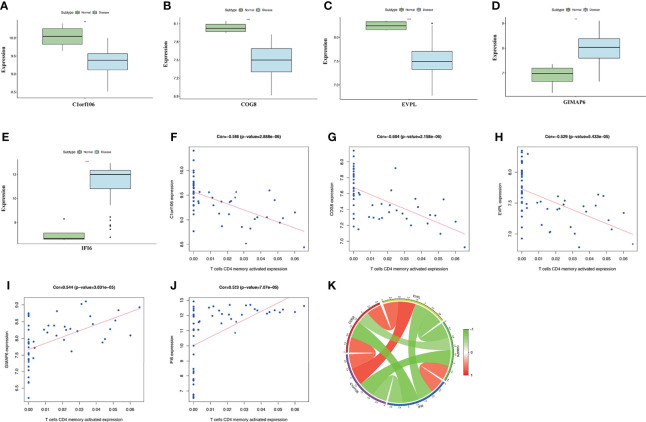
**(A–E)** The expression level of the five key genes between the DM and normal groups (^**^ *p* < 0.01; ***p < 0.001; *p* < 0.05 were considered significantly different). **(F–J)** The correlation analysis between key genes and T-cell CD4 memory activated expression. The correlation coefficients and *p*-values were shown at the top of the graphs (*p* < 0.01 indicated the significant correlation). **(K)** The Circos diagram depicts Pearson correlations between key genes. The red color represents positive correlation, and the green color represents negative correlation.

The expression of the key genes in damaged skin tissues of DM patients was analyzed, and the results suggested that, compared with the normal group, the expression of *C1orf106*, *COG8*, and *EVPL* was lower, and the expression of *IFI6* and *GIMAP6* was higher in the DM group (**Figures 6A–E**). The correlation analysis of the key genes suggested that *C1orf106* presented a significantly positive correlation with *COG8* and *EVPL*, *IFI6* presented a significantly positive correlation with *GIMAP6*, and the remaining genes were pairwise negatively correlated (**Figures 6F–J**). These mechanisms require further exploration.

The correlation analysis between the expression levels of the key genes and CD4^+^ T cells indicated that the expression of *C1orf106*, *COG8*, and *EVPL* was significantly negatively correlated with the expression of T-cell CD4 memory activated. Whereas, the expression of *GIMAP6* and *IFI6* presented a remarkably positive correlation (**Figure 6K**). The results presented above suggested the expression levels of the key genes in DM all promoted the infiltration of CD4^+^ T cells. To evaluate the changes of the key gene expression levels in the lesional skin before and after treatment of DM patients, we analyzed the GSE193276 dataset and found that the *EVPL* expression level was significantly increased and the *IFI6* expression level was significantly decreased after treatment (**Supplementary Figure S1**). It indicated that *EVPL* and *IFI6* may not only be related to the infiltration of CD4^+^ T cells but also are more likely to participate in the progression of DM compared with the other three key genes.

### GSEA Analysis Results of Key Genes

**Figure 7:**
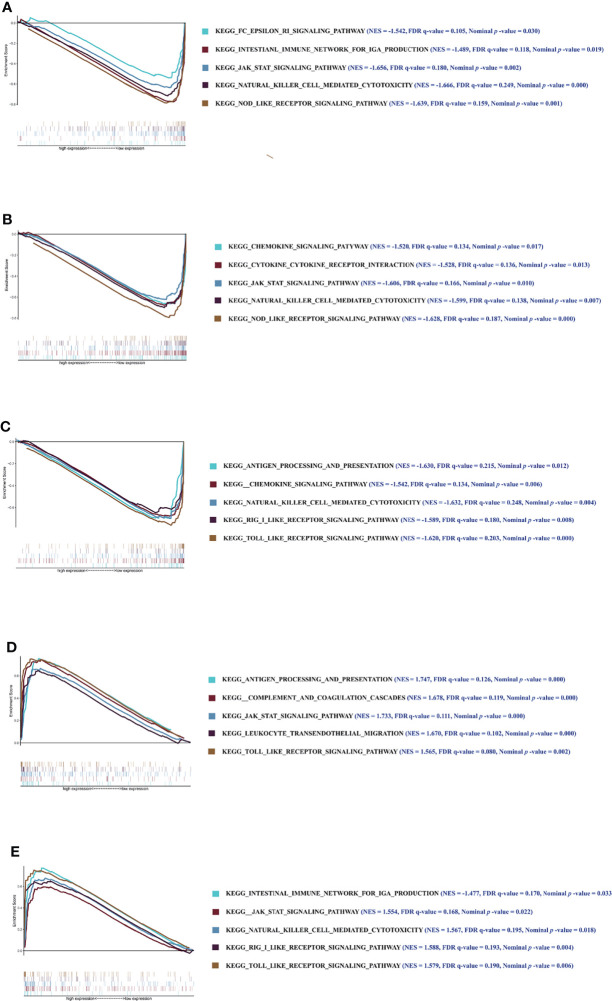
The results of GSEA. Enrichment analysis of pathway and KEGG-involved key genes. **(A–E)** Graphs respectively show the GSEA results of *C1orf106*, *COG8*, *EVPL*, *GIMAP6*, and *IFI6* genes. Each graph includes 2 parts. The first part is line graph of enrichment score (ES), the horizontal axis represents ranked gene set, and the vertical axis represents running ES (NES represents normalized ES). The score at the peak of the line graph is the ES for that gene set. The position of gene set was marked by the vertical line in the second part (The absolute value of the NES >1, the false-positive rate (FDR) *q*-value <0.25, and the nominal *p-*value <0.05 were considered as the significantly enriched pathways).

Furthermore, to investigate the underlying molecular mechanisms of the key genes affecting the CD4^+^ T-cell infiltration in DM, we conducted enrichment analysis for the 5 key genes involved in signaling pathways. GSEA analysis showed that the key genes involved in many immune-related pathways were significantly enriched. The Janus Kinase/Signal transducers and activators of transcription (JAK/STAT) signaling pathway, NOD-like receptor signaling pathway, natural killer cell-mediated cytotoxicity, etc., were significantly enriched in the low-expression group of C1orf106. The chemokine signaling pathway, NOD-like receptor signaling pathway, cytokine–cytokine receptor interaction, etc., were significantly enriched in the low-expression group of *COG8*. The TOLL-like receptor signaling pathway, antigen processing and presentation, chemokine signaling pathway, etc., were significantly enriched in the low-expression group of *EVPL*. Leukocyte transendothelial migration, the JAK/STAT signaling pathway, complement and coagulation cascades, etc., were significantly enriched in the high-expression of *GIMAP6*. The JAK/STAT signaling pathway, retinoic acid inducible-gene I (RIG-I)-like receptor signaling pathway, intestinal immune network for IgA production, etc., were significantly enriched in the high-expression group of *IFI6* (**Figure 7**). The above results suggest that *C1orf106*, *COG8*, *EVPL*, *GIMAP6*, and *IFI6* genes may affect the infiltration of CD4^+^ T cells in DM through diverse immune-related pathways.

### Enrichment Analysis for Transcription Factors of Key Genes

**Figure 8:**
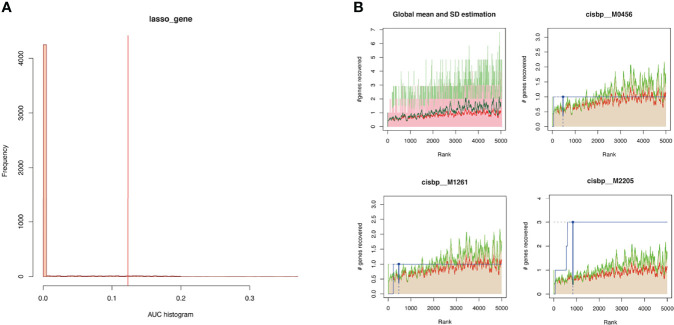
**(A)** Histogram of AUC. The first step to assess the overrepresentation of each motif for key genes is to calculate the AUC. The red vertical line represents the significance level that motifs with a greater AUC than the significance level are regarded as significant motifs. **(B)** The recovery curve for a few motifs. The red line represents the global mean of recovered curve of motifs, and the green line represents mean ± standard deviation. Motifs greater than mean ± standard deviation were regarded statistically significant. The blue line represents the recovered curve of the current motif. The motif cisbp_M2205 was significantly enriched in key genes (*C1orf106*, *EVPL*, and *IFI6*), while cisbp_M0456 and cisbp_M1261 did not reach the significance level.

Gene modules are composed of coexpression genes, suggesting potential coregulatory mechanisms such as multiple transcription factors. In view of this, the enrichment analysis of transcription factors was performed (**Figure 8**). The enrichment analysis includes three steps: (1) The cumulative recovery curve was used for enrichment analysis; (2) the annotation of Motif-TF; and (3) the significant gene selection. The enrichment analysis results indicated that the highest NES of Motif-TF annotated as cisbp_M2205 was 7.46, and the 3 key genes (*C1orf106*, *EVPL*, and *IFI6*) were enriched in this motif, indicating that the transcription binding domain was the master regulatory factor for key genes *C1orf106*, *EVPL*, and *IFI6*. Meanwhile, all the enriched motifs and corresponding transcription factors for the 5 key genes are displayed in **Supplementary Table S7**.

### Correlation Analysis Between Key Genes and Disease-Regulating Genes

**Figure 9:**
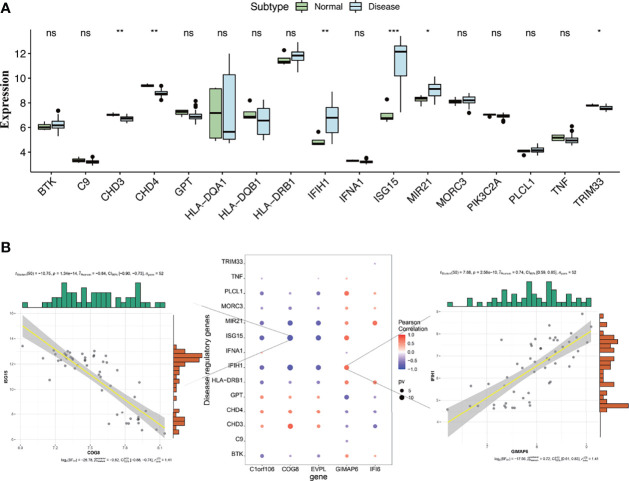
**(A)** Differential analysis of disease-regulating genes. Regulatory genes of *CHD3*, *CHD4*, *IFIH1*, *ISG15*, *MIR21*, and *TRIM33* showed significantly different expression between the DM and normal control groups. Compared to normal control, *CHD3*, *CHD4*, and *TRIM33* were lowly expressed in the DM group, while *IFIH1*, *ISG15*, and *MIR21* were highly expressed (^*^ *p* < 0.05, ^**^ *p* < 0.01, ^***^ *p* < 0.001, ns, no significance; *p* < 0.05 were considered significantly different). **(B)** The correlation analysis of key genes and differential regulatory genes. The first plot indicates *COG8* was significantly negatively correlated with *ISG15*, the second pot indicates visualization of Pearson correlation between regulatory genes and key genes, and the third plot indicates *GIMAP6* was significantly positively correlated with *IFIH1*. The *P*earson correlation coefficients and *p*-values were shown at the top of the graphs (*p* < 0.01 indicated the significant correlation).

These genes involved in the development and progression of diseases are called disease-regulating genes. Regulatory genes identified by the GeneCards database were most likely involved in the development of DM and were analyzed for differential expression between DM groups and normal groups. The results showed that regulatory genes chromodomain-helicase-DNA-binding protein (*CHD)* 3, *CHD4*, interferon induced with helicase C domain 1 (*IFIH1)*, interferon-stimulated gene 15 (*ISG15)*, microRNA 21 (*MIR21)*, and tripartite motif-containing 33 (*TRIM33*) had significant differences in expression between the two groups (**Figure 9A**). The correlation analysis of the key genes and differential regulatory genes indicated that *COG8* was significantly negatively correlated with *ISG15* (*P*earson correlation coefficient = -0.84, *p* = 1.34e−14) and *GIMAP6* was significantly positively correlated with *IFIH1* (*P*earson correlation coefficient = 0.74, *p* = 2.56e−10) (**Figure 9B**). The above results suggested that *COG8* and *GIMAP6* may take part in the regulation of *ISG15* and *IFIH1* on DM, respectively.

### Construction of the lncRNA-miRNA-mRNA (ceRNA) Network

According to the ceRNA hypothesis, miRNAs negatively regulate the expression of their target mRNAs by binding to response elements in target mRNAs, while lncRNAs inhibit miRNAs’ negative regulation on target mRNAs through acting as molecular sponges (28, 29). We constructed a lncRNA-miRNA-mRNA (ceRNA) network. Based on the mRNAs of the 5 key genes, miRNAs of the key genes were predicted reversely by applying the miRcode database, and 396 miRNA-mRNA interaction pairs were obtained. Afterward, we used the DIANA-LncBase database to identify reversely lncRNAs, and 8,769 lncRNA-miRNA interaction pairs were obtained. A total of 18,598 lncRNA-miRNA-mRNA relation pairs were obtained, involving 52 miRNAs and 3,835 lncRNAs (**Supplementary Table S8**). Eventually, the ceRNA network was constructed, using the Cytoscape software to visualize this network (**Supplementary Figure S2**). The top 5 connectivity degree of lncRNAs were CTC-459F4.3, KCNQ1OT1, AC006548.28, MIR6818, and RP3-323A16.1.

## Discussions

The definitive pathogenesis of DM has not been fully determined, but it is widely believed to be an autoimmune response that environmental factors trigger in genetically susceptible individuals. The most favorable evidence in support of immune-mediated pathogenesis is the presence of multiple immune cell infiltration and autoantibodies in muscle biopsies of DM patients. Studies have shown that T lymphocytes are related to the pathogenesis of DM and the disease activity, but the specific mechanism remains unclear (30). CD4^+^ T cells are the most considerable immune infiltrating cells in the skin, muscle, and bronchoalveolar lavage fluid of DM patients, so it is crucial to explore potential therapeutic targets based on CD4^+^ T cells. However, no studies so far have systematically screened biomarkers correlated with CD4^+^ T-cell immune infiltration and evaluated their value in the immune infiltration process of DM. CD4^+^ T cells are related to the area and the severity of skin lesions, and some skin manifestations are associated with the prognosis of patients, indicating that CD4^+^ T cells play an important role in the pathogenesis and prognosis of DM skin damage. This study first identified the key genes associated with CD4^+^ T-cell infiltration in skin biopsy samples of DM patients by integrating bioinformatics analysis and then validated the better model performance in a muscle biopsy dataset from DM patients to further search for potential targeted genes involved in the pathogenesis of DM.

The WGCNA analysis suggested that the brown module was the most correlated with the level of CD4^+^ T-cell infiltration within the WGCNA coexpression network. Gene enrichment analysis identified the brown module was highly correlated with immunity. The 5 key genes related to the CD4^+^ T-cell infiltration level, namely *C1orf106*, *COG8*, *EVPL*, *GIMAP6*, and *IFI6*, have been identified by the LASSO regression model. Moreover, the expression levels of the 5 key genes could promote CD4^+^ T-cell infiltration in DM patients. This model was well validated in the DM muscle biopsy dataset, indicating this predictive model may also assess the level of CD4^+^ T-cell infiltration of the muscle involvement in DM. These results indicated that the 5 key genes are factors affecting the immune infiltration of CD4^+^ T cells in DM patients.

*C1orf106*, also known as innate immunity activator (*INAVA*), is located on chromosome 1 and encodes the C1orf106 protein (31). The C1orf106 protein is an epithelial junction protein that maintains the junction of the functional epithelial cells through the direct interaction with adhesins and is essential for maintaining the integrity of the intestinal epithelial barrier (31). The polymorphism of *C1orf106* is a susceptibility factor for inflammatory bowel disease (IBD), and the decrease of C1orf106 protein expression could lead to the loss of intestinal mucosa barrier integrity, resulting in increased susceptibility to IBD (32). Human macrophages carrying the *C1orf106*-risk allele rs7554511 reduced the expression of C1orf106 protein, decreasing the NOD2 signaling and the secretion of cytokines initiated by pattern recognition receptors (PRR) (33). The impaired T-cell function and Th cell differentiation were observed in the absence of NOD2 (34). We found that the NOD-like receptor signaling pathway was enriched in *C1orf106* low expression, but the result seems different from that of the previous study (33), which may be one of the reasons why *C1orf106* low expression promotes the infiltration of CD4^+^ T cells in DM patients. Our study suggested that other molecular mechanisms such as the enrichment of the JAK/STAT signaling pathway may be related to the low expression of *C1orf106*, promoting CD4^+^ T-cell infiltration. Studies have shown that methylation changes of CD4^+^ T cells affect the polarization of CD4^+^ T cells, which may be the pathogenesis of psoriasis (35, 36). Zhou et al. found that the single nucleotide polymorphisms (SNPs) rs2853953 in *C1orf106* may mediate the genetic risk of psoriasis through DNA methylation (37). The above studies indicated that the SNPs in the *C1orf106* gene may affect CD4^+^ T-cell polarization through methylation. Overexpression of *C1orf106* was associated with invasive breast cancer and its poor prognosis and could be used as a novel marker to predict the aggressiveness and prognosis of breast cancer (38). In addition, 2 SNPs (rs442905 and rs59457695) in the *C1orf106* gene and protein expression levels could be used to predict the therapeutic effect of infliximab in patients with Crohn’s disease (39). Based on the above, it appears particularly interesting to further explore the relationships between the *C1orf106* gene and DM, *C1orf106*, and CD4^+^ T cells.

The *COG8* gene encodes the COG8 protein which is involved in intracellular membrane transport and protein glycosylation (40). The dysfunction of the COG complex interferes with the glycosylation of proteins by affecting the separation of glycosyltransferase (41). Receptors related to T-cell differentiation, such as CD4, CD8, cytotoxic T-lymphocyte-associated protein 4 (CTLA-4), and Notch receptors, are glycoprotein receptors whose expression and function depend on normal glycosylation (42). Previous studies suggested that glycosylation has a certain regulatory effect on T-cell–mediated diseases such as multiple sclerosis (MS), systemic lupus erythematosus (SLE), and IBD (43–46). The silencing of COG8 could enhance the expression of immune-related genes (47). Chemokines are the key drivers of inflammation. Their functions include not only appealing leukocytes to the inflammation sites but also activating adhesion molecules to permit leukocyte extravasation (48). Liu et al. found, after silencing the *COG8* gene, the mean fluorescence intensity (MFI) of the chemokine receptor C-X-C motif chemokine receptor 4 (CXCR4) on the surface of CD4^+^ T cells infected with human immunodeficiency virus (HIV)-1 had a slight increase (49). Dendritic cells induce germinal center (GC) CD4^+^ T-cell migration by upregulating CXCR4 on the surface of CD4^+^ T cells (50). Schjetne found CXCR4 was one of the extraordinary targets for Ab-mediated delivery of Ag for major histocompatibility complex, class II (MHC-II) presentation and could promote CD4^+^ T-cell proliferation (51). Moreover, CXCR4 was related to idiopathic CD4^+^ T lymphocytopenia (52). Our results also indicated a chemokine signaling pathway was associated with *COG8* low expression. A worthy of consideration is that *COG8* may promote CD4^+^ T-cell infiltration in the skin by multiple pathways, including the chemokine signaling pathway and glycosylation. Nevertheless, since there are no studies between *COG8* and DM, it requires further experimental validation. *COG8* is a potential prognostic biomarker in kidney renal clear cell carcinoma (KIRC), and the KIRC patients with low expression of *COG8* showed worse progression-free survival and disease-free survival (53). Whether *COG8* can be used as a prognostic marker in DM remains to be explored.

The EVPL protein is encoded by the *EVPL* gene and is a member of the plakin protein family, predominantly expressed in the skin, esophagus, and other tissues. The cornified envelope is indispensable for the skin barrier function, while envoplakin, periplakin, and involucrin jointly form the protein scaffolds of the cornified envelope (54). Sevilla et al. found that combined loss of the cornified envelope protein (EPI^−/−^) could not only damage the epidermal barrier but also increase CD4^+^ T-cell infiltration and decrease γδ T cells, changing the composition of T-cell subsets (54). The expression levels of cytokines and chemokines increased, and the responsiveness of lymphoid stress surveillance intensified (55). Shen et al. found that the EVPL protein expression was downregulated in mouse atopic dermatitis skin lesions, the skin injury was significantly improved, and the expression of EVPL in skin lesions was upregulated after resveratrol treatment (56). Similarly, our results also found the expression of *EVPL* in skin lesions was also increased. It has been shown that *EVPL*, as a novel biomarker in metastatic melanoma, can be used to predict the poor prognosis of patients with metastatic melanoma (57). Although there are presently no studies about *EVPL* related to the pathogenesis of DM, combined with the evidence presented above, we thought that *EVPL* deficiency may lead to skin damage in patients with DM, which is most likely related to CD4^+^ T-cell infiltration.

The GTPase of immunity-associated proteins (GIMAPs) are a family of genes thought to be involved in the development, signaling, and apoptosis of lymphocytes, having an essential role in immune system homeostasis (58). *GIMAP6* is located on chromosome 7q36.1 and encodes the GIMAP6 protein. GIMAP6 protein is an antiapoptotic protein associated with T cells and a member of the GIMAP family that is dominantly expressed in CD3^+^ T lymphocytes (59). GIMAP6 protein can act on the regulation of the activation and apoptosis of peripheral T cells to maintain the homeostasis of peripheral T cells, and the dysregulation of T-lymphocyte homeostasis is closely linked to autoimmune diseases (58, 60). The apoptosis of CD4^+^ T lymphocytes was accelerated and the number of CD4^+^ T cells was significantly reduced in the peripheral blood of the *GIMAP6* gene-deficient mice (60). Genetic association studies have shown that the *GIMAP* gene is related to autoimmune diseases such as SLE, Behcet’s syndrome, type I diabetes (61–63). An increase in GIMAP6 protein level enhances the survival rate of activated T cells by increasing the resistance to cell death induced by genotoxic restimulation and activation (59). Thereby, in autoimmune diseases, *GIMAP6* may promote the T-cell–mediated immune response immunity by regulating T-cell activity. Combined with our results that *GIMAP6* expression was significantly upregulated in DM, we speculated that *GIMAP6* might contribute to regulating the development, activation, and apoptosis of CD4^+^ T cells. *GIMAP6* has been recognized as a prognostic biomarker in head and neck squamous cell carcinoma, breast cancer, and female lung adenocarcinoma and as a predictor of response to immunotherapy in lung adenocarcinoma (64–66). We are looking forward to furthering studies exploring the relationship between *GIMAP6* and CD4^+^ T cells in DM.

*IFI6* is an interferon-stimulated gene (*ISG*), and its expression is highly induced by IFN-α (67). IFI6 protein, encoded by *IFI16*, participates in the immune system response to type I interferon (IFN-I) by activating the JAK/STAT signaling pathway engaged in immune regulation and antiapoptosis (67). It has been clear that the pathogenesis of DM is related to IFN-I, especially IFN-β, which is highly expressed in T cells in DM cutaneous lesions, and the IFN-I pathway is highly active in DM skin lesions (14). The infiltration of CD4^+^ T lymphocytes increased in DM skin lesions and strongly expressed IFN-β and IFN-γ (9). In juvenile dermatomyositis (JDM) patients, the disease activity relates to the IFN-I and IFN-II scores (68). Sustained IFN responses mediated by continuous stimulation of antigen-presenting cells are implicated in a variety of autoimmune diseases, and the resulting activation of T and B cells may be accountable for the generation of autoantibodies (69). Studies implied that the mRNA expression of *IFI6* was remarkably increased in the muscle tissue of DM patients (70). IFI6 was highly expressed in SLE, psoriasis, and type I diabetes and was also able to forecast the treatment response of rheumatoid arthritis patients to tocilizumab (71–73). The IFN-I pathway also mediates the upregulation of hundreds of *ISG* through the JAK/STAT signaling pathway (74). Our study also showed that the JAK/STAT signaling pathway was enriched in the high expression of *IFI6*. Therefore, *IFI6* and IFN-I may form a positive feedback loop. IFN-α and IFN-β can activate CD4^+^ T cells, and IFN-α can mediate the differentiation of CD4^+^ T cells towards Th1 cells (75–77). This evidence indicated that the highly expressed *IFI6* may promote the infiltration of CD4^+^ T cells and regulate the body’s immune function to participate in the occurrence and development of DM by being involved in the IFN-I signaling pathway.

Wong et al. found that a large group of genes involved in T cells and IFN-induced genes were overexpressed in skin lesion biopsies of patients with DM, especially in active skin lesions such as *ISG15* and *IFIH* (78). *ISG15* is an IFN-1 induced gene and encodes ISG15 protein, which is highly upregulated in muscle, blood, and skin of DM patients (78–80). *ISG15* is widely considered to be a regulatory gene in DM and may regulate the pathogenesis of DM by driving injury mechanisms of myofibers and capillary DM (81). *IFIH1* is also named melanoma differentiation–associated gene 5 (*MAD5*), and the IFIH1 protein encoded by this gene acts as a cytoplasmic sensor that recognizes viral double-stranded RNA and then triggers transcription of genes encoding type I interferons (24). The IFIH1 protein is considered a specific autoantigen target of DM, and the anti-MDA5 antibody is highly expressed in DM (82, 83). It is speculated that the virus may trigger the overproduction of IFN-I, thereby promoting the development of anti-MDA5–associated DM. Patients with anti-MDA5–associated DM have unique cutaneous manifestations and an increased risk of rapidly progressive interstitial lung disease (RP-ILD), leading to high mortality (24). The biological pathways of *ISG15* and *IFIH1* include T-cell activation, antigen processing, complement activation, etc. (78). The percentage of CD4^+^CXCR4^+^ T cells in the peripheral blood of idiopathic inflammatory myopathy–related ILD (IIM-ILD) patients was significantly increased. CD4^+^CXCR4^+^ T cells are a novel biomarker of IIM-ILD, which could predict disease severity and prognosis (84). Our study found that the key genes *COG8* and *GIMAP6* were related to the upregulation of *ISG15* and *IFIH* in DM skin lesions, suggesting that *COG8* and *GIMAP6* may be involved in the occurrence and development of DM and may mediate the infiltration of CD4^+^ T cells in DM through regulatory genes. Further studies on the relationship between the key genes, disease-regulating genes, and CD4^+^ T cells may better reveal the pathogenesis of DM.

In the end, we constructed the ceRNA regulatory network to gain insight into the upstream regulatory sites of the key genes. However, this study has some limitations. Only bioinformatics methods were used for key gene screening and verification, which is in the prediction stage. Additional clinical samples, *in vivo* and *in vitro* experiments, and functional studies are needed to validate the prediction results.

## Conclusions

In summary, we found that the 5 key genes, *C1orf106*, *COG8*, *EVPL*, *GIMAP6*, and *IFI6*, were associated with the CD4^+^ T-cell infiltration in lesional skin tissues of DM, and the prediction model constructed based on the 5 key genes may better also predict the level of CD4^+^ T-cell infiltration in damaged muscle tissues of DM. Therefore, these key genes could be underlying diagnostic markers and immunotherapy targets for DM. This study may provide a novel perspective for further understanding the mechanism and new orientations for the treatment of DM based on CD4^+^ T cells.

## Data Availability Statement

The datasets presented in this study can be found in online repositories. The names of the repository/repositories and accession number(s) can be found in the article/**Supplementary Material**.

## Author Contributions

LQL conceived the ideas of the study. PH designed methods and analyzed the data and wrote the draft paper. LT and LZ helped download and analyze the data. YR and HP contributed analysis tools and displayed the arrangement of figures. YX and JX participated in the refinement of design methods and the analysis of data. DM gave some suggestions for the drafting of the paper. LQL and LJL reviewed and revised the paper. All authors contributed to the article and approved the submitted version.

## Funding

This project was supported by China National Natural Scientific Foundation grants (No. 81873762 and 81501039), Science and Technology Department of Hunan Province Funds (No. 2022SK2032 and 2018SK2069), and Health Select Commission of Hunan Province Funds (No. B20180311).

## Conflict of Interest

The authors declare that the research was conducted in the absence of any commercial or financial relationships that could be construed as a potential conflict of interest.

## Publisher’s Note

All claims expressed in this article are solely those of the authors and do not necessarily represent those of their affiliated organizations, or those of the publisher, the editors and the reviewers. Any product that may be evaluated in this article, or claim that may be made by its manufacturer, is not guaranteed or endorsed by the publisher.

## Supplementary Material

Supplementary Figure S1 The expression levels of the key genes before and after treatment in the GSE193276 validation set (**p* < 0.05, ***p* < 0.01, ns: no significance; *p* < 0.05 were considered significantly different).

Supplementary Figure S2 The visualization results of the lncRNA-miRNA-mRNA(ceRNA) network of key genes.

Supplementary Table S1 GSE46239 data.

Supplementary Table S2 GSE142807 data.

Supplementary Table S3 GSE1551 data.

Supplementary Table S4 GSE193276 data.

Supplementary Table S5 WGCNA results and Gene names in each module.

Supplementary Table S6 Representative genes of Functional Enrichment analysis.

Supplementary Table S7 The Enrichment Analysis results of Transcription Factors of key genes.

Supplementary Table S8 The lncRNA-miRNA-mRNA relation pairs.

## Abbreviations

AUC, area under the curve; CADM, clinically amyopathic dermatomyositis; CB2R, cannabinoid type 2 receptor; ceRNA, competing endogenous RNA; CHD3, chromodomain-helicase-DNA-binding protein 3; CIBERSORT, cell type identification by estimating relative subsets of RNA transcripts; COG8, component of oligomeric Golgi complex 8; Cor, correlation coefficient; CTLA-4, cytotoxic T-lymphocyte–associated protein 4; CXCR4, C-X-C motif chemokine receptor 4; DM, dermatomyositis; EVPL, envoplakin; ES, enrichment score; FDR, false-positive rate; GC, germinal center; GEO, Gene Expression Omnibus; GIMAPs, GTPases of immunity-associated proteins; GIMAP6, GTPases of immunity-associated protein family member 6; GO, Gene Ontology; GPL, GEO platforms; GSEA, Gene Set Enrichment Analysis; HIV-1, human immunodeficiency virus 1; IBD, inflammatory bowel disease; IFI6, interferon-alpha inducible protein 6; IFIH1, interferon induced with helicase C domain 1; IFN, interferon; IFN-I, type I interferon; IL, interleukin; ILD, interstitial lung disease; IIM-ILD, idiopathic inflammatory myopathy–related ILD; INAVA, innate immunity activator; ISG, interferon-stimulated gene; ISG15, interferon-stimulated gene 15; JAK/STAT, Janus kinase/signal transducers and activators of transcription; JDM, juvenile dermatomyositis; KEGG, Kyoto Encyclopedia of Genes and Genomes; KIRC, kidney renal clear cell carcinoma; LASSO, least absolute shrinkage and selection operator; lncRNA, long noncoding RNA; MAAs, myositis-associated autoantibodies; MSAs, myositis-specific autoantibodies; MAD5, melanoma differentiation–associated gene 5; ME, module eigengene; MFI, mean fluorescence intensity; MHC-II, major histocompatibility complex, class II; mRNA, messenger RNA; miRNA, MicroRNA; MIR21, microRNA 21; MM, module membership; MS, multiple sclerosis; NES, normalized enrichment score; PBMCs, peripheral blood mononuclear cells; RIG-I, retinoic acid inducible-gene I; ROC curve, receiver operating characteristic curve; PRR, pattern recognition receptors; RP-ILD, rapidly progressive interstitial lung disease; SLE, systemic lupus erythematosus; SNPs, single nucleotide polymorphisms; TFs, transcription factors; Th, T helper; TOM, topological overlap matrix; TRIM33, tripartite motif-containing 33; WGCNA, weighted gene coexpression network analysis.

## Glossary

**Table:**

AUC	area under the curve
C1orf106	chromosome 1 open reading frame 106
CADM	clinically amyopathic dermatomyositis
CB2R	cannabinoid type 2 receptor
ceRNA	competing endogenous RNA
CHD3	chromodomain- helicase- DNA- binding protein 3
CIBERSORT	cell type identification by estimating relative subsets of RNA transcripts
COG8	component of oligomeric Golgi complex 8
Cor	correlation coefficient
CTLA-4	cytotoxic T-lymphocyte–associated protein 4
CXCR4	C-X-C motif chemokine receptor 4
DM	dermatomyositis
EVPL	envoplakin
ES	enrichment score
FDR	false-positive rate
GC	germinal center
GEO	Gene Expression Omnibus
GIMAPs	GTPases of immunity-associated proteins
GIMAP6	GTPases of immunity-associated protein family member 6
GO	Gene Ontology
GPL	GEO platforms
GSEA	Gene S e t Enrichment An a l y s i s
HIV-1	human immunodeficiency virus 1
IBD	inflammatory bowel disease
IFI6	interferon-alpha inducible protein 6
IFIH1	interferon induced with helicase C domain 1
IFN	interferon
IFN-I	type I interferon
IL	interleukin
ILD	interstitial lung disease
IIMILD	idiopathic inflammatory myopathy–related ILD
INAVA	innate immunity activator
ISG	interferon-stimulated gene
ISG15	interferon-stimulated gene 15
JAK/STAT	Janus kinase/signal transducers and activators of transcription
JDM	juvenile dermatomyositis
KEGG	Kyoto Encyclopedia of Genes and Genomes
KIRC	kidney renal clear cell carcinoma
LASSO	least absolute shrinkage and selection operator
lncRNA	long noncoding RNA
MAAs	myositis-associated autoantibodies
MSAs	myositis-specific autoantibodies
MAD5	melanoma differentiation–associated gene 5
ME	module eigengene
MFI	mean fluorescence intensity
MHC-II	major histocompatibility complex class II
mRNA	messenger RNA
miRNA	microRNA
MIR21	microRNA 21
MM	module membership
MS	multiple sclerosis
NES	normalized enrichment score
PBMCs	peripheral blood mononuclear cells
RIG-I	retinoic acid inducible-gene I
ROC curve	receiver operating characteristic curve
PRR	pattern recognition receptors
RP-ILD	rapidly progressive interstitial lung disease
SLE	systemic lupus erythematosus
SNPs	single nucleotide polymorphisms
TFs	transcription factors
Th	T helper
TOM	topological overlap matrix
TRIM33	tripartitemotif-containing 33
WGCNA	weighted gene coexpression network analysis
