# Supplementary figures and images for: Identification of Biomarkers Associated With CD4+ T-Cell Infiltration With Gene Coexpression Network in Dermatomyositis
Source: Front Immunol. 2022 May 30;13:854848. doi: 10.3389/fimmu.2022.854848 (PMC9196312; doi:10.3389/fimmu.2022.854848)

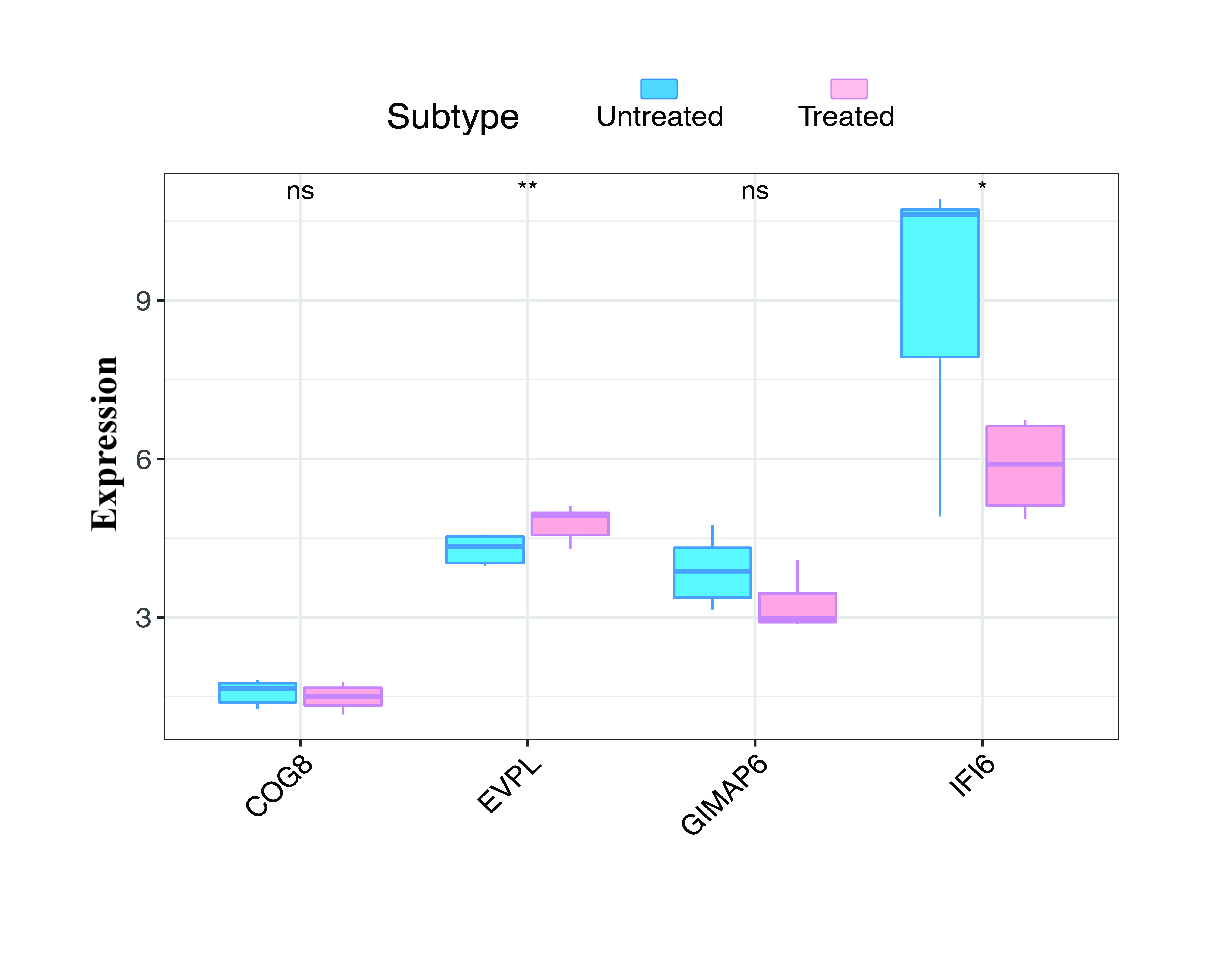

Supplement: Supplementary Figure S1 — The expression levels of the key genes before and after treatment in the GSE193276 validation set (*p < 0.05, **p < 0.01, ns: no significance; p < 0.05 were considered significantly different). [file Image_1.jpeg]

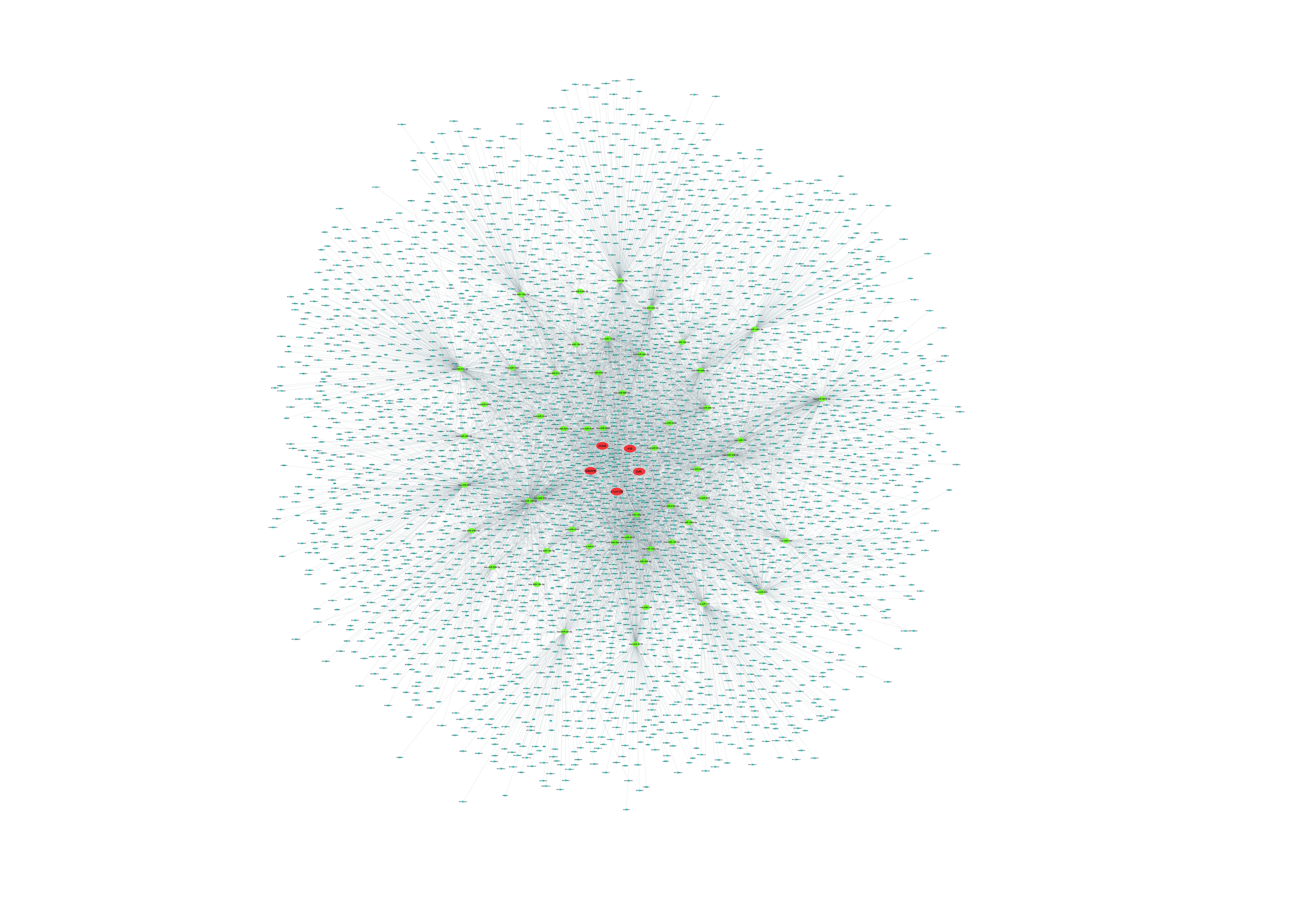

Supplement: Supplementary Figure S2 — The visualization results of the lncRNA-miRNA-mRNA(ceRNA) network of key genes. [file Image_2.jpeg]
